# Railway track settlement patterns and control measures for multi-tunnel construction underneath a station track group: a case study

**DOI:** 10.1038/s41598-024-64916-6

**Published:** 2024-06-24

**Authors:** Pinpin Li, Feng Lu, Haiyun Huang, Wenge Qiu

**Affiliations:** 1 Key Laboratory of Transportation Tunnel Engineering, Ministry of Education, Southwest Jiaotong University, Chengdu, 610031 China; 2 School of Emergency Science, Xihua University, Chengdu, 610039 China; 3 Chengdu Tianyou Tunnelkey Co., Ltd., Chengdu, 610031 China

**Keywords:** Ground settlement, Track deformation, Construction method, Numerical simulation, Civil engineering, Computational science

## Abstract

This paper is based on the proximity engineering project of the Baishiyi tunnel group passing under the Chongqing West Station track group. Considering the train load and the spatial relationship of the tunnel and track groups, the settlement patterns, horizontal displacement, and differential settlement of the tunnel–strata–tracks system during the excavation process are studied through theoretical calculations and numerical simulation methods. The results indicate that the tunnel vault, strata, and track settlement deformation patterns are similar. Throughout the tunnel construction process, the tracks underwent uplift, settlement, and eventually stabilized. The settlement trough formed by the excavation of the three tunnels below the track group has an impact range of 25–145 m. Between 35 and 75 m, the differential settlement of the double track gradually increases with excavation. As the tunnel face reaches 75 m, the track differential settlement gradually converges and tends to stabilize. To minimize the impact of underpass tunnel construction on track groups, it is recommended to use a combination of full-section hole grouting and surface reinforcement grouting for ground reinforcement. Additionally, optimizing the construction parameters, including the step length and primary support closure time, and strengthening the locking anchor can further reduce the impact.

## Introduction

China has seen a significant increase in infrastructure construction in recent years, and numerous multi-tunnel proximity projects have been developed underneath existing railway lines, high-speed railway bridges, and train stations^1–3^. The dense rail area of a station is an important part of the railway infrastructure, which usually includes many switches and track crossings and is a bottleneck of the traffic capacity of the station^4^. As the operating railway in dense railway areas has strict displacement requirements, tunnel construction can pose risks to train operational safety, increase maintenance costs, and affect the efficiency of train operations. For instance, the water conveyance tunnel passing beneath the vehicle section of Hangzhou North Station caused deformation of the ballast on the Shanghai–Kunming Railway^5^; the Caochang Street Tunnel passing beneath Zhengzhou Railway Station led to extensive subgrade settlement^6^. Ensuring operational safety and reducing the impact of multi-tunnel construction beneath operating railways has become an urgent engineering challenge that needs to be addressed.

The results of field tests and numerical results show that shallow tunnel excavation easily causes obvious ground subsidence^7^. Moreover, the trainloads of multiple parallel tracks will be superimposed on the crossing area, which further increases the challenge of track deformation control^8^. It is essential to analyze the impact of tunnel construction on the operation of the overhead railway and to determine the appropriate measures that should be taken during the construction of the tunnel. During the construction of the Wuhan subway tunnel, researchers studied the influence of railway subgrade settlement. When the tunnel excavation surface was approximately 6 m away from the centreline of the subgrade, the existing subgrade experienced obvious settlement, and the disturbance of the first tunnel construction was particularly significant^9^. Tunnel construction has an obvious influence on the smoothness of the subgrade and railway tracks. The overhead rails will follow the strata and subgrade during settlement, and the greater the settlement of the ground and subgrade, the more obvious the rail unevenness becomes^10^. In soft soil areas, the deformation of the overlying strata takes a longer time, and the subsequent settlement accounts for a significant proportion of the total settlement. It is also necessary to monitor the railway settlement over an extended period. When constructing a tunnel in soft soil strata or shallowly buried strata, it is usually necessary to reinforce the foundation of the railway subgrade^11,12^. During the construction of subway systems in Beijing, Guangzhou, and London, the issue of settlement in tunnel railway stations and railway lines was encountered^13–15^. Researchers have conducted studies on settlement control measures. Engineering experience has shown that a rational excavation method combined with grouting reinforcement can control the longitudinal and transverse settlements, as well as uneven settlement, caused by tunnel construction in existing railways. Currently, a significant amount of research has been conducted on the proximity effects of tunnels and the control of railway deformation. However, there is still a lack of studies on the deformation patterns of track groups, differential settlement changes, and control strategies in areas with dense railway tracks underneath train stations where multiple tunnels are constructed close.

Based on the Chongqing Baishiyi tunnel group beneath the track group of Chongqing West Station, this paper proposes the adoption of the double-side drift method with core soil to reduce the impact of tunnel construction on ground settlement and track deformation. Taking into account the train loads and the spatial relationship with the tunnel, this study investigates the settlement patterns of the tunnel–strata–track system, the horizontal displacement of the tracks, and the differential settlement during tunnel construction by integrating theoretical calculations with numerical simulations. The applicability and rationality of this method are analyzed, and engineering measures are recommended to reduce the impact of the tunnel on the track group. This research aims to provide a reference for impact analyses of multi-tunnel projects passing beneath railway infrastructure.

## Engineering background

### Project overview

**Figure 1:**
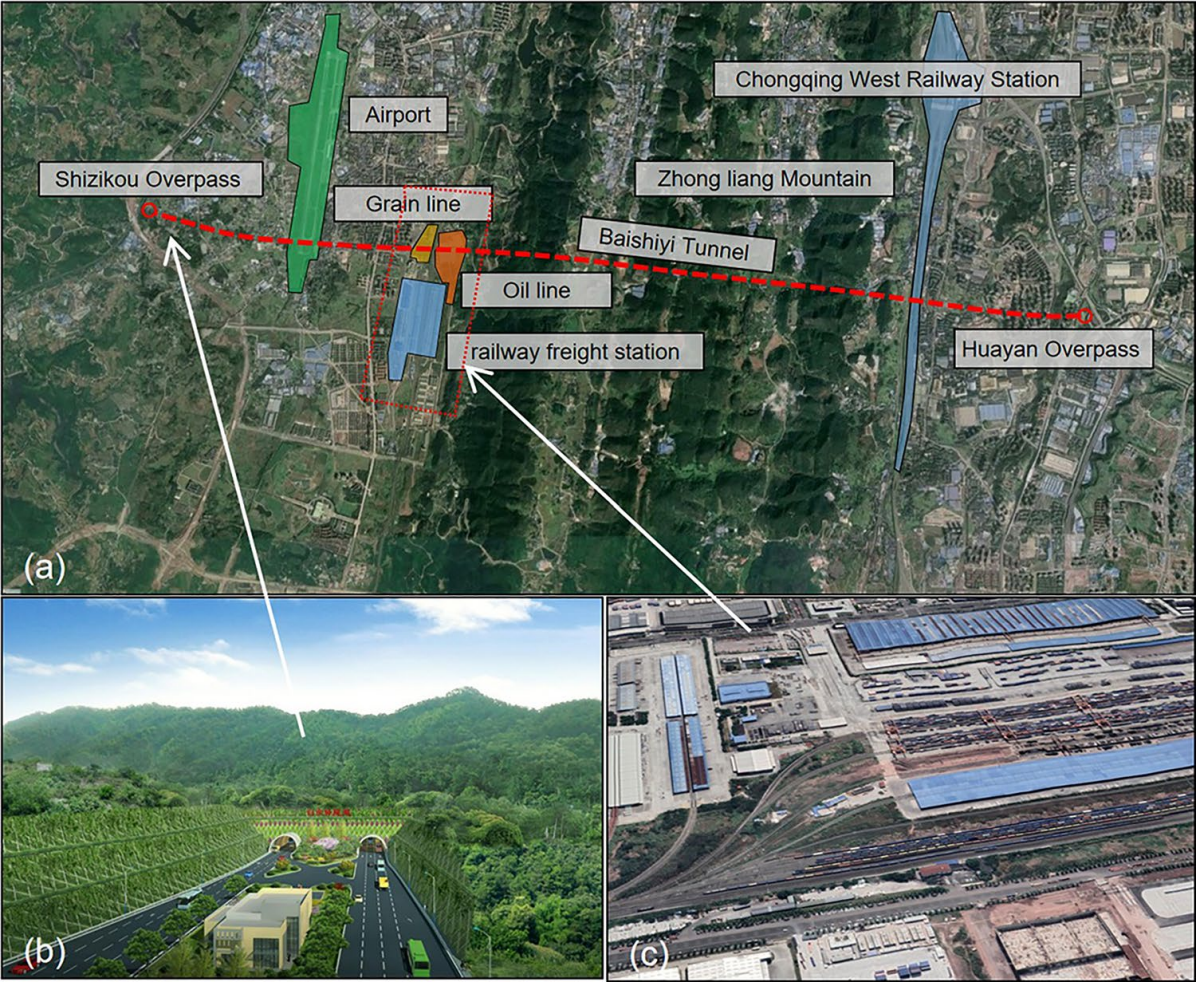
Geographic map of the project location: (**a**) the project location map (This figure were generated used by the software Google Earth, the URL link is https://earth.google.com), (**b**) the tunnel entrance, (**c**) the rail group distribution.

The Baishiyi tunnel of Chongqing starts at the Shizikou Interchange of the expressway, passes through Zhongliang Mountain, and ends at the Huayan Interchange. The western side of the Baishiyi tunnel passes the Chongqing-Qianghan freight railway of the Chongqing South Railway Station. Railway settlement control is required to meet high standards. To minimize the impact of tunnel construction on ground settlement, excavation methods and construction plans that can meet railway operating conditions are needed. Due to the limitations of site and operation conditions, conventional measures such as d-beam and stratum grouting reinforcement cannot be carried out. The project location is depicted in Fig. 1 on the geographic map.

**Figure 2:**
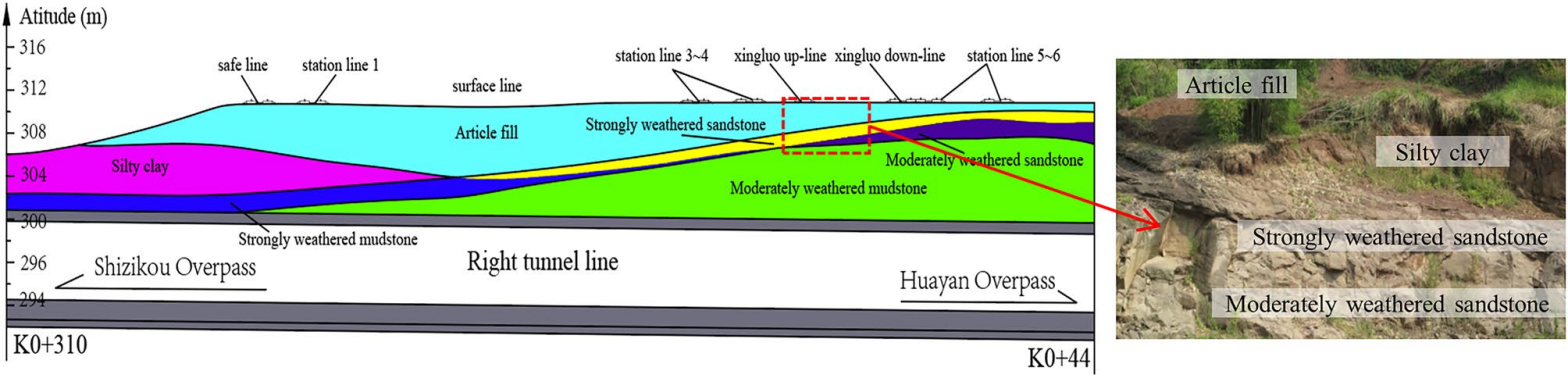
Stratigraphic distribution of the profile and the bedrock outcrops.

The project section features two main outcrops: the Shaximiao Group (J2s) and the Xintiangou Group (J2x) of the Middle Jurassic. The Holocene series consists mainly of miscellaneous fill, silt, and silty clay. The Middle Jurassic Shaximiao Group strata is primarily composed of interbedded sandstone and mudstone, while the Xintiangou Group strata are mainly composed of sandstone, mudstone, siltstone, and shale. Figure 2 displays the stratigraphic distribution of a longitudinal geological profile. Table 1 presents the mechanical parameters of each rock stratum.

**Table 1:** Tunnel lining support parameter scheme (unit: mm).

Lining type	Primary lining			Secondary lining	Preliminary support measures	
	Bolt	Steel arch	Shotcrete	Reinforced concrete	Steel arch	Deformation allowance
Baishiyi tunnel	Ф25 grouting anchor rods, length 4500. Circular spacing of 800, longitudinal spacing of 600	HK240b full ring steel arch @ 600, Ф 8 steel mesh sheets (150 × 150)	C30, thickness 300	C40, thickness 650	Ф108 advance pipe shed with a ring distance of 400	80
Auxiliary Tunnel B	Ф25 grouting anchor rod, length 4500. Circular spacing of 800, longitudinal spacing of 600	I20b full ring steel arch @ 600 Ф8 steel mesh sheets (150 × 150)	C30, thickness 280	C30, thickness 550	Ф108 advance pipe shed with a ring distance of 400	60

### Spatial relationships

**Figure 3:**
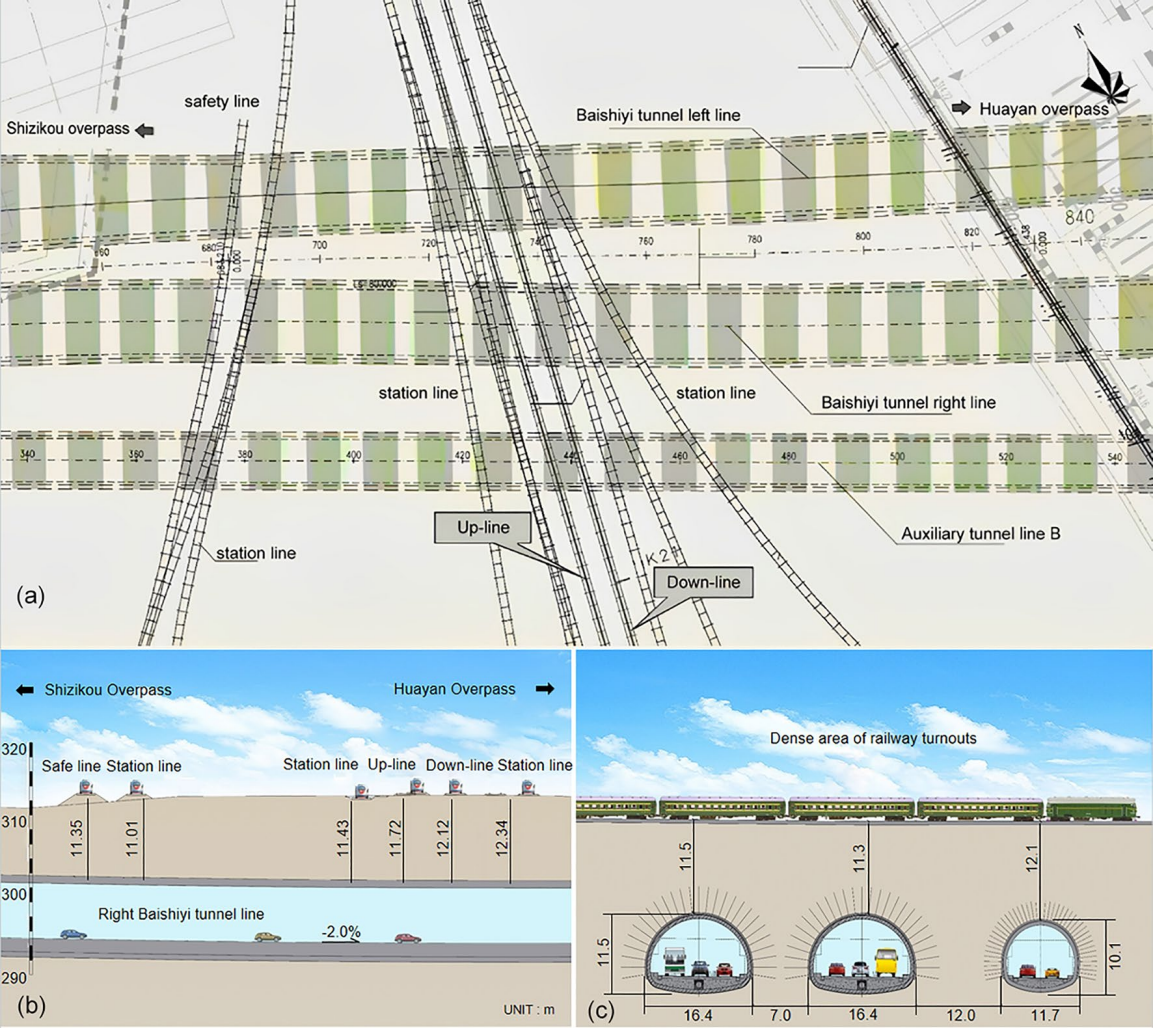
The spatial relationship between the railway and the Baishiyi tunnel: (**a**) planar relationship, (**b**) cross-section relationship, (**c**) longitudinal section relationship.

The Baishiyi tunnel group traverses under the rail cluster of the Chongqing South Railway Station, encompassing ten railway lines, including the Grain Special Line and Xingluo Line. Figure 3 illustrates the spatial relationship between the Baishiyi tunnel group and the overlying railway track group. The tunnel's left line runs through the railway area K0 + 340 ~ K0 + 500, while the right line runs through the railway area K0 + 340 ~ K0 + 520. Additionally, the auxiliary line B tunnel passes through the railway K0 + 360 ~ K0 + 5605 (Fig. 3a). The minimum depth of the left line tunnel is approximately 11.35 m, and that of the right line tunnel is approximately 11 m (Fig. 3b). The left and right lines are approximately 7 m apart, while the distance between the right line and auxiliary line B is approximately 12 m. The Baishiyi tunnels have diameters of approximately 16.4 m, and the auxiliary line B tunnel has a diameter of approximately 11.7 m (Fig. 3c).

### Tunnel excavation method

Due to the large size of the tunnel design section and poor ground conditions, the double-side drift method is advantageous for controlling the deformation of the surrounding rock and surface settlement. Additionally, the upper step of the core soil helps stabilize the tunnel face. Therefore, in this case, a tunnel crossing a dense section of railway tracks is constructed using the double-side drift method with reserved core soil^16^. The auxiliary line B tunnel section is constructed using the step method and features a composite lining. The primary support includes grouting anchor, steel arch frame, and shotcrete, while the secondary lining is made of reinforced concrete. Table 1 lists the specific support parameters.

**Figure 4:**
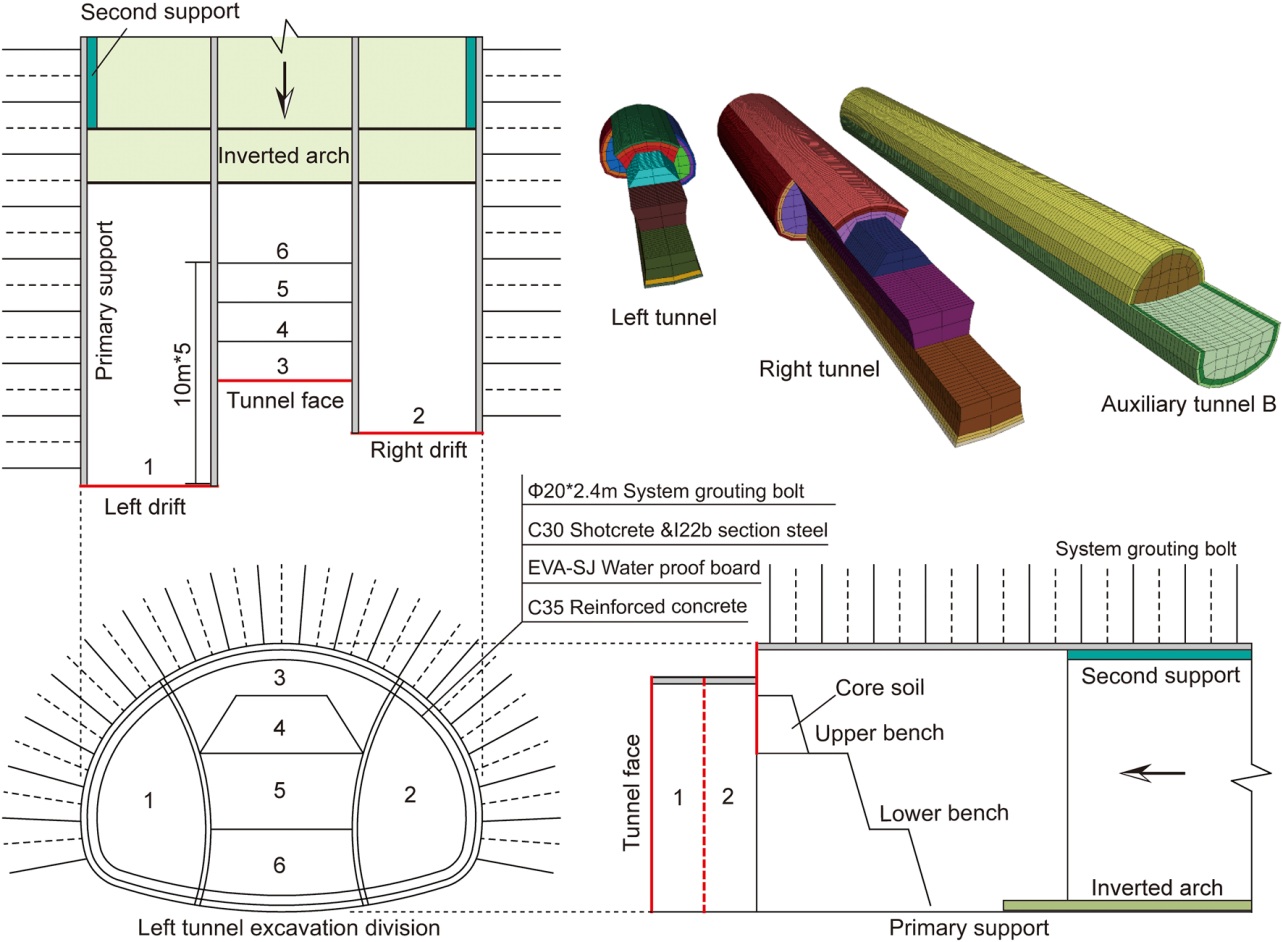
Excavation process of the double-side drift method.

The tunnel construction process involves excavating the left side of Guide Pit 1 and installing initial and temporary support, followed by excavation of the right side of Guide Pit 2 with the same support installation. The next step is the excavation of upper step 3, with the same support installation, followed by the excavation of the core soil. The excavation process involves the middle step, followed by the lower step, with the primary support being stabilized after the application of the uplift arch. Finally, the tilting arch is applied, and the secondary lining is poured. The construction process of the double-side drift method with core soil is shown in Fig. 4. The tunnel support parameters are listed in the table.

## Engineering risks and control standards

Considering the engineering geology and site investigation of the Baishiyi tunnel beneath the railway station with dense tracks, the project faces three principal risks:

1. Shallow-buried tunnel excavation risk: the shallow overburden of the tunnel with a minimum depth of 11.35 m and the closely spaced distance between tunnels (7 m, less than one diameter) poses a significant excavation safety challenge. Employing the double-side drift method, the 45 m interval between tunnel faces may lead to high risks due to the proximity of construction.
2. Geotechnical instability risk: The tunnel passes through the stratum of miscellaneous fill, silty soil, and silty clay, characterized by extensive weathering fractures, which compromises tunneling conditions. The potential for water seepage in sandstone layers adds to the risks of instability, strata displacement, and leakage.
3. Railway operational risks: As the tunnel construction is close to 10 railway tracks and numerous forks, the influence of deformation needs to be strictly controlled. The heavy axle load of freight trains intensifies the deformation of the crossing area, which may endanger railway safety.

### Risk control factors

The influence of tunnel underpasses on stratum and railway track settlement is mainly affected by four factors: tunnel construction, geology, track type, and train loading^17–19^ (Table 2). Various construction methods disturb the strata differently, and the strength and effectiveness of tunnel support structures also affect the strata and track settlement. The track structure produces varying stress distributions in the soil layer below it. The stiffness of the ground track affects the transfer of loads to the strata, which in turn affects the settlement. The train weight, velocity, and operating frequency are the primary variables affecting train loading. Heavy trains exert greater pressure on the ground strata. High-speed trains may generate higher dynamic loads on the track. The frequency of train operation directly affects the cumulative settlement. Therefore, these factors must be considered in the tunnel design and construction, and appropriate measures must be taken to control ground settlement to ensure project safety and stability.

**Table 2:** Control factors of surface subsidence and track deformation.

Control factors	Specific parameters
Excavation method	NATM/TBM/cut-and-cover method/freezing method
Support structure	Steel arch, grid steel frame, temporary inverted arch, system anchor rod, and concrete formwork design parameters
Tunnel geometric parameter	Tunnel diameter, tunnel burial depth, spacing between adjacent tunnels, and step length
Ground geological condition	Rock mass strength and deformation modulus, rock joint and fissure parameters, formation permeability, groundwater conditions
Train type and load	Train operating speed, train axle load, train operating frequency
Track type	Ballastless versus ballasted track, track weight

### Track settlement control criteria

The unevenness of an existing railway line is measured in two ways: (a) the horizontal deviation, which refers to the relative height difference of the deviation between the two strands of the rail surface; (b) the vertical deviation, which refers to the height difference along the direction of the line before and after the location of the deviation. Exceeding the allowable limit of deviation can cause the vehicle to shake and result in uneven wear and tear of the rail due to uneven forces on the two strands of the rail^17^. The negative impact of unevenness on the front and rear of the vehicle is directly proportional to the depth of a pothole and inversely proportional to its length. This can pose a serious safety risk to line operations. The deformation of track structures is influenced by various factors, such as railway speed, type of railway fasteners, and railway maintenance conditions. Deformation is measured by indicators such as the deformation amount and deformation rate, which encompasses settlement and uplift. The control standard is based on the permissible deviation of track static geometry in the China Railway Line Repair Rules standard^20^, which primarily considers deformation. Table 3 displays the governing values of static track deformation in the railway control specification.

**Table 3:** Management value of track static deformation in railway control code.

Item (mm)	*V*_*max*_ > 160 km/h			160 km/h ≥ *V*_*max*_ > 120 km/h			*V*_*max*_ ≤ 120 km/h		
	A	B	C	A	B	C	A	B	C
Rail spacing	+ 2	+ 4	+ 6	+ 4	+ 6	+ 8	+ 6	+ 7	+ 9
	− 2	− 2	− 4	− 2	− 4	− 4	− 2	− 4	− 4
Horizon difference	3	5	8	4	6	8	4	6	10
Vertical difference	3	5	8	4	6	8	4	6	10
Rail track direction	3	4	7	4	6	8	4	6	10

*V*_*max*_ is the maximum railway speed; A indicates normal railway operation; B indicates frequent railway maintenance; C indicates temporary railway repair.

## Numerical modelling

### Tunnel modelling

**Figure 5:**
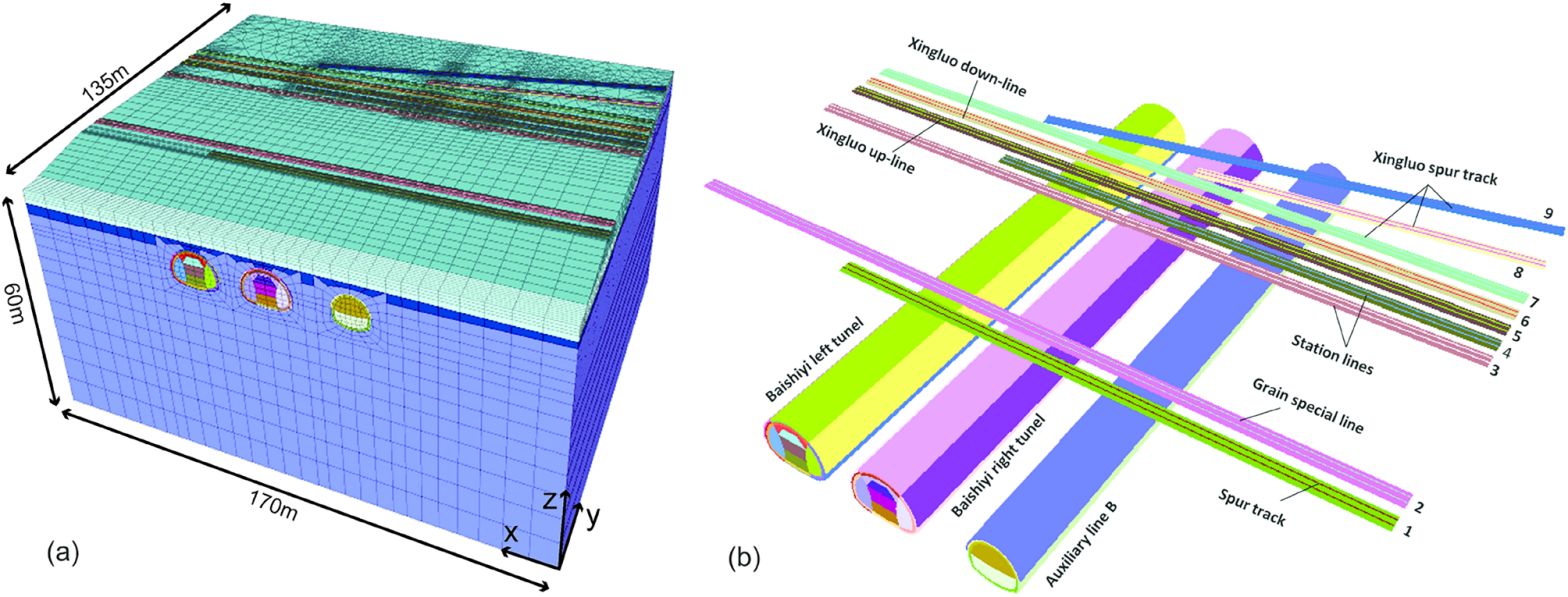
Numerical calculation model: (**a**) stratigraphic-structural models, (**b**) tunnel and track structures.

To analyze the ground settlement and track deformation during the construction of multiple tunnels and to evaluate the applicability of the double-sidewall tunneling method in the project, a 3D numerical model was established using FLAC3D finite difference simulation software. The model has an overall size of 60 m × 135 m × 170 m. The station track group is oriented along the Y-axis, while the railway tunnel is bored along the X-axis, as illustrated in Fig. 5a. The positional relationship of the tunnel and track in the model is shown in Fig. 5b. The model's bottom is subjected to fully fixed constraints, while the top is designated a free boundary. The remaining four faces are subjected to normal sliding constraints. The model is divided into 270 parts along the longitudinal direction, with each cell having a length of 0.5 m. The mesh around the tunnel is denser in the cross-sectional direction, while it is sparser farther away. The model comprises 258,484 nodes and 284,242 elements. The soil layer was modeled using the modified Mohr–Coulomb model, while the lining, steel support, and grouting layers were modeled using the elastic model. Table 4 shows the calculated parameters for the modeled soil layers and supporting structures.

**Table 4:** Mechanical parameters of the surrounding rocks, rails, and track beds.

Material type	Density (kg/m^3^)	Elastic modulus (MPa)	Poisson’s ratio	Frictional angle (°)	Cohesion (kPa)
Article fill	2000	1	0.40	30.00	0
Silty clay	1960	15	0.36	13.15	22.2
Strongly weathered mudstone	2470	400	0.36	27.00	110
Primary support	1950	28,000	0.3		
Secondary support	2150	31,500	0.3		
Railroad bed	2240	5800	0.3	50	300
43# Rail track	2397	210,000	0.3	–	–
50# Rail track	2450	225,000	0.3		
60# Rail track	2550	235,000	0.3		

The main tunnel was excavated using the double-side drift method with reserved core soil, and the auxiliary line B tunnel was excavated using the step method. The excavation sequence of the tunnels was left line–right line–auxiliary line B from left to right. The excavation faces were spaced longitudinally by 10 m in each tunnel. The distance between different tunnels was 3 times the diameter of the tunnel, which was 45 m. Figure 5 shows the spatial relationship between the tunnel model and the structure.

### Trainload arrangement

**Figure 6:**
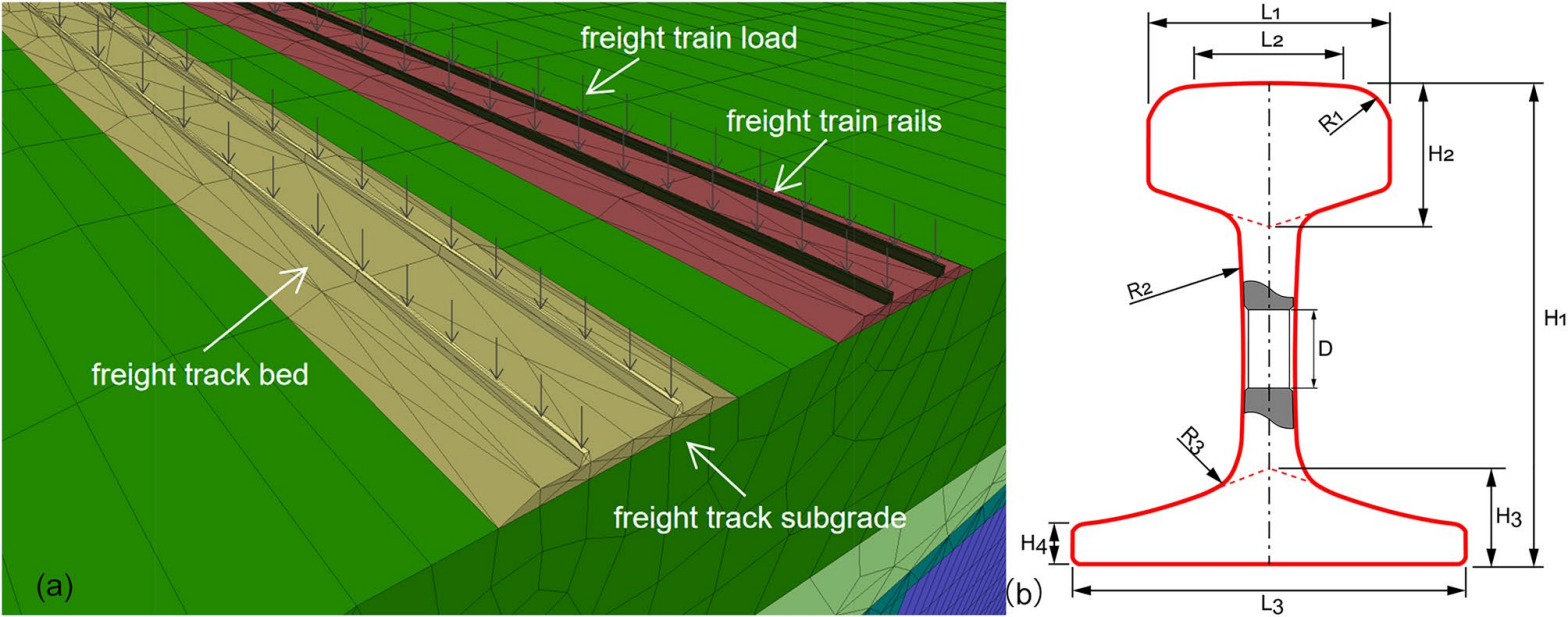
Simplified model of a railway track: (**a**) rail numerical model, (**b**) track cross-section design drawing.

The track pressure is determined by using a continuous beam model with elastic point support to simulate the train load. The principle of superposition is applied to calculate the joint action of the wheel load under the pressure of the sleeper. It is assumed that the pressure is uniformly distributed within the range of the length of the effective support. According to the model of a continuous beam with elastic point support, a single wheel load generates a reaction force on a single rail sleeper. The train load is simplified as a series of constant forces moving uniformly on the rail, and the dynamic effect of the interaction between the vehicle and the track is reflected by the impact coefficient. Therefore, the reaction force on this rail sleeper under the simultaneous action of multiple wheel loads is calculated. Assuming that the pressure of the rail sleeper is uniformly distributed within the effective support length, the top surface pressure of the roadbed can be calculated. The train load is considered to be a uniform load in the calculation (shown in Fig. 6a). Before applying the train equivalent load to the track surface, it is necessary to balance the geostatic stress. The model should have a large redundancy along both the longitudinal and transverse directions to reduce the boundary effect on the calculation results.

1 $$ R_{i} = \frac{Pkd}{2}\varphi \left( {x_{i} } \right) $$

2 $$ \varphi \left( {x_{i} } \right) = e^{ - k\left| x \right|} \left( {\cos k\left| x \right| + \sin k\left| x \right|} \right) $$

3 $$ R = \frac{{P\left( {1 + \alpha + \beta } \right)kd}}{2}\mathop \sum \limits_{i = 1}^{n} \varphi \left( {x_{i} + vt} \right) $$

4 $$ \sigma = R/\left( {be} \right) $$

where P represents the wheel load, $k$ represents the rail base and rail rigidity ratio coefficient, $d$ represents the spacing of the sleeper, and $x$ represents the distance between the wheel load and the sleeper. $\alpha$ represents the speed coefficient, $\beta$ represents the bias load coefficient, $b$ represents the width of the sleeper (which is fixed at 0.28 m), and $e$ represents the effective support length of the sleeper (which is fixed at 1.1 m).

Rail stiffness plays an important role in load dispersion, improving track integrity and stability, and has a significant impact on track settlement. There are various types of railway rails used in China. For light-load railway lines, 43 kg/m rails are suitable, while 50 kg/m rails are commonly used for freight lines. For high-speed and heavy-load railways, 60 kg/m rails are usually preferred. The International Union of Railways (UIC) classifies rails into three main types: UIC33, UIC54, and UIC60. Figure 6b shows the rail profile design and Table 5 shows the specific rail parameters for different sizes according to GB/T 2585-2021.

**Table 5:** The Chinese railway standard rail type parameters (mm).

Railway track type	Cross-sectional area (mm^2^)	Horizontal moment of inertia (cm^4^)	Vertical moment of inertia (cm^4^)	H1	H2	H3	L1	L2	L3
43#	5677	1479.6	257.2	140	42	27	70	46	114
50#	6555	2025.4	374.2	152	42	27	70	46	132
60#	7740	3215.2	523.5	176	48.5	30.5	70.8	50.7	150

## Results and discussion

During the construction of railway tunnels beneath existing roads and railways, the primary effect is the loss of the overlying soil layer of the tunnel, which leads to the settlement and deformation of the foundations of the existing roads and railways. The displacement mechanisms for both the railway foundation and the ground surface due to new tunnel construction are fundamentally similar, with differences mainly related to the existing railway's track structure and train loads.

### Track longitudinal settlement pattern

**Figure 7:**
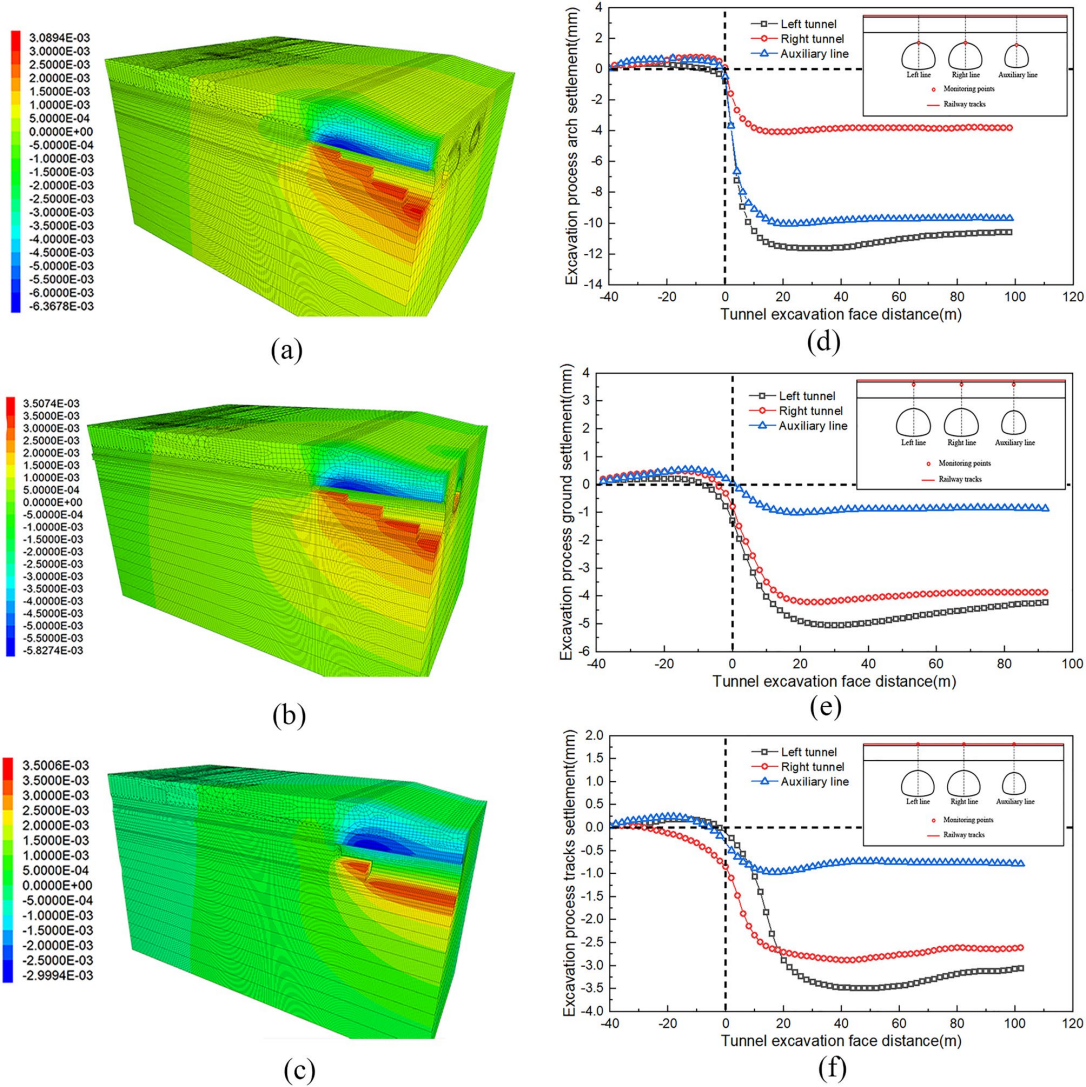
Vertical settlement contour map and change curve.

To study the deformation of the track group during the tunnel excavation process, the deformation of the excavated tunnel and railway under dedicated railway #1 is monitored and analyzed. The minimum distance between the dedicated railway #1 and the tunnel is 10.96 m, hence it is considered that the intersection section of the dedicated railway 1 with the tunnel is the most unfavorable. Figure 7 shows the deformation of the vault, surface, and track of railway #1. The variation in displacement during tunnel face excavation and the settlement contour diagram during tunnel excavation are also monitored. Table 6 shows the deformation of the tunnel vault and railway tracks. The x-axis zero point corresponds to the excavation of the middle platform at the top of the double-sidewall headings directly beneath the dedicated railway line. The horizontal axis of each curve starts at the excavation of the upper step of the middle guide of the corresponding tunnel to the special railway right below. The left tunnel arch reaches a maximum of 11.9 mm, while the right tunnel arch reaches a maximum of 10.7 mm. The maximum settlement of the auxiliary line B tunnel arch is 4.6 mm, which is close to the maximum permissible settlement of 12 mm. These results indicate that the tunnel chamber can be self-stabilized after excavation. However, settlement and deformation may exceed certain limits, and it is necessary to monitor settlement during construction. Furthermore, upon comparing the curve of changes in the vault with the advancement of the palm face and the corresponding contour diagram, it is evident that the settlement of the 10 m vault after the excavation of the upper step of the tunnel guide accounts for 90% of the final settlement value. This indicates that settlement control during the excavation of the vault is crucial, and it is necessary to apply this control for primary support promptly. During the construction process of the tunnel passing beneath the railway, the railway track structure experienced a process of initial uplift, followed by settlement, and finally reached a stable condition. After the tunnel face crossed under the railway line at a distance of 20 m, the influence on track settlement gradually decreased due to the distance from the excavation face, and the settlement became essentially stable.

**Table 6:** Deformation of tunnel vault and railway tracks.

Construction stage	Vault deformation (mm)			Track deformation (mm)		
	Left line	Right line	Auxiliary line B	Left line	Right line	Auxiliary line B
Left excavation face arrival	− 1.26	− 0.95	− 0.61	− 0.44	− 0.39	− 0.29
Right excavation face arrival	− 10.63	− 9.64	− 4.28	− 3.22	− 2.86	− 1.03
Auxiliary line B excavation faces arrival	− 11.94	− 10.77	− 4.68	− 6.88	− 5.69	− 1.56
Auxiliary line B excavation face passes through 20 m	− 11.91	− 10.59	− 4.59	− 6.64	− 5.43	− 1.51
Finish	− 11.75	− 10.51	− 4.54	− 6.25	− 5.28	− 1.52

The track settlement above the left tunnel has a maximum value of 6.88 mm, while the maximum value above the right tunnel is 5.69 mm. The maximum track settlement above the auxiliary ramp is 1.56 mm. When the excavation of the right-line tunnel reached directly beneath the dedicated railway line 1, and the face of the left-line tunnel advanced to a position 45 m ahead, the railway track once again experienced subsidence.

### Track lateral settlement pattern

**Figure 8:**
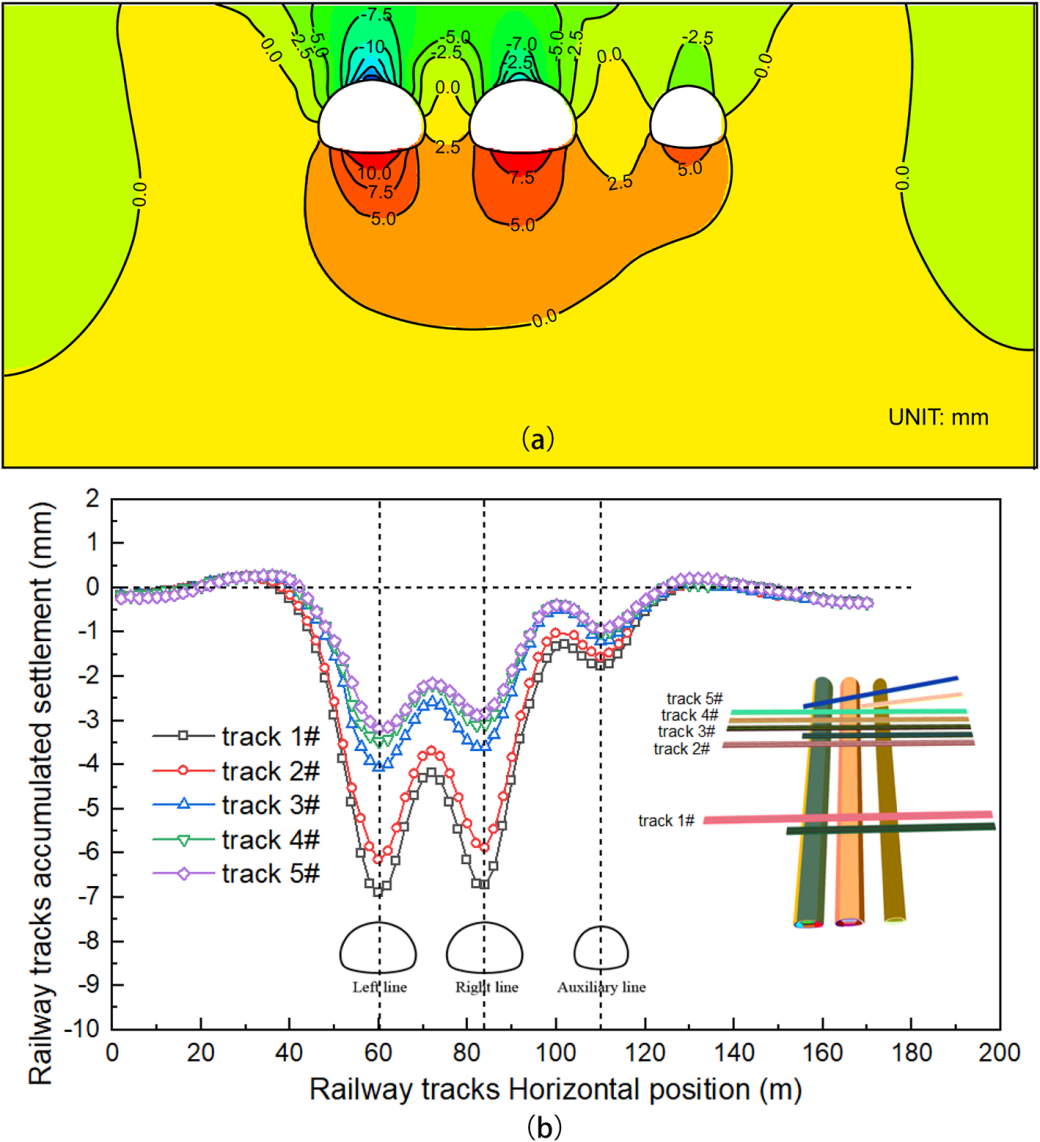
Lateral settlement pattern: (**a**) Horizontal cumulative settlement contour map, (**b**) Tracks accumulated settlement curve.

Upon completion of the excavation of the Baishiyi tunnel, both the ground and railway tracks experienced significant settlement and deformation. The maximum settlement of the ground surface was 7.8 mm, while the maximum settlement of the track was 6.8 mm, which occurred at the monitoring point of Special Line Railway 1 directly above the tunnel of the left line. The stability of the surrounding rock is poor due to the thin overlying rock layer in this section of the tunnel. To prevent track settlement from exceeding the limit after tunnel excavation, corresponding support measures must be taken, and monitoring must be strengthened. Additionally, the Baishiyi tunnel group has a clear distance of 7 m, and the final settlement of the left tunnel is partly due to the excavation of the left tunnel. Figure 8a shows the settlement displacement contour diagram of the section where Special Line Railway 1 is located. Figure 8b shows the cumulative settlement and deformation of the track after the excavation is completed. The track serial numbers 1–5 represent the railway line above the tunnel mileage from small to large, respectively: the Special Line Railway turnoff, the Special Line Railway, the Xingluo Line turnoff, and the Xingluo Line upwards and downwards lines. The double tunnel beneath the surface settlement curve was of the ‘w’ type. Additionally, auxiliary line B had a small surface settlement groove. The surface settlement caused three holes under the railway track group, as shown in Fig. 8a. The cumulative lateral deformation of the track is shown in Fig. 8b. When ground settlement occurs, the track will follow suit. The settlement of each layer increases sequentially from top to bottom, and the magnitude of the ground settlement directly affects the degree of track settlement.

### Correlations between the track type and differential settlement

**Figure 9:**
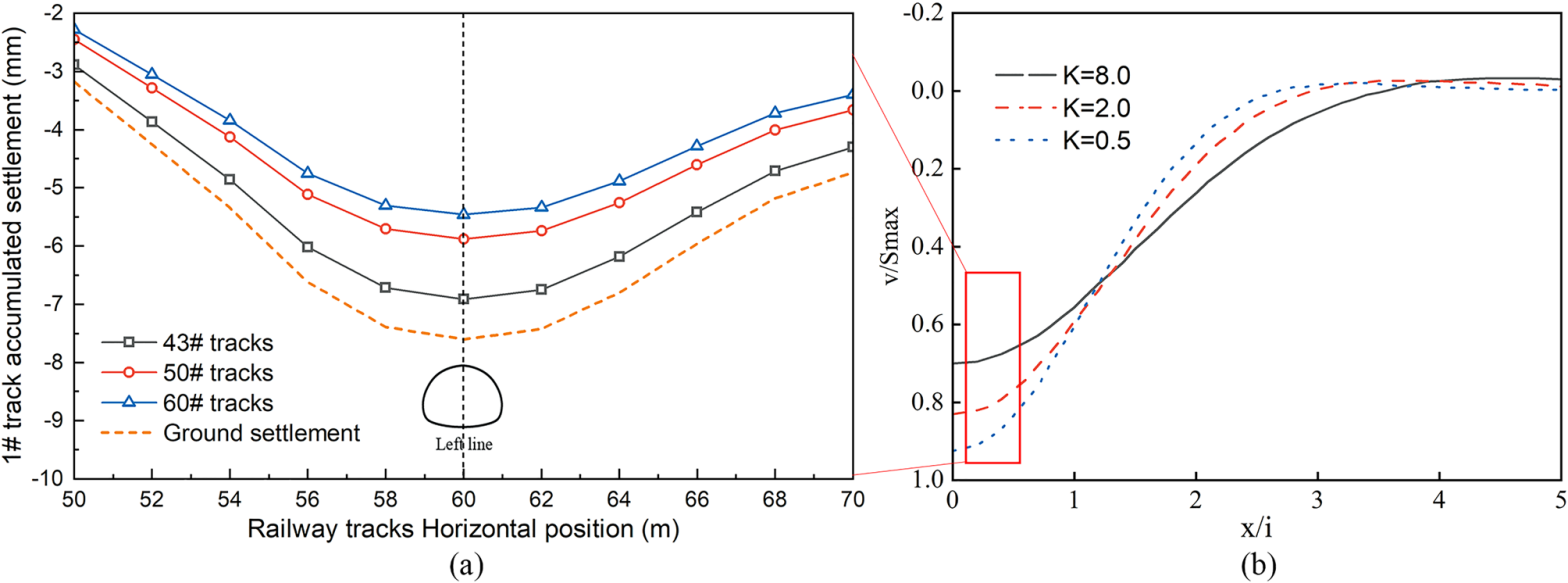
Settlement of different track types: (**a**) accumulated settlement of different track types, (**b**) effect of different track stiffnesses on settlement^21^.

**Figure 10:**
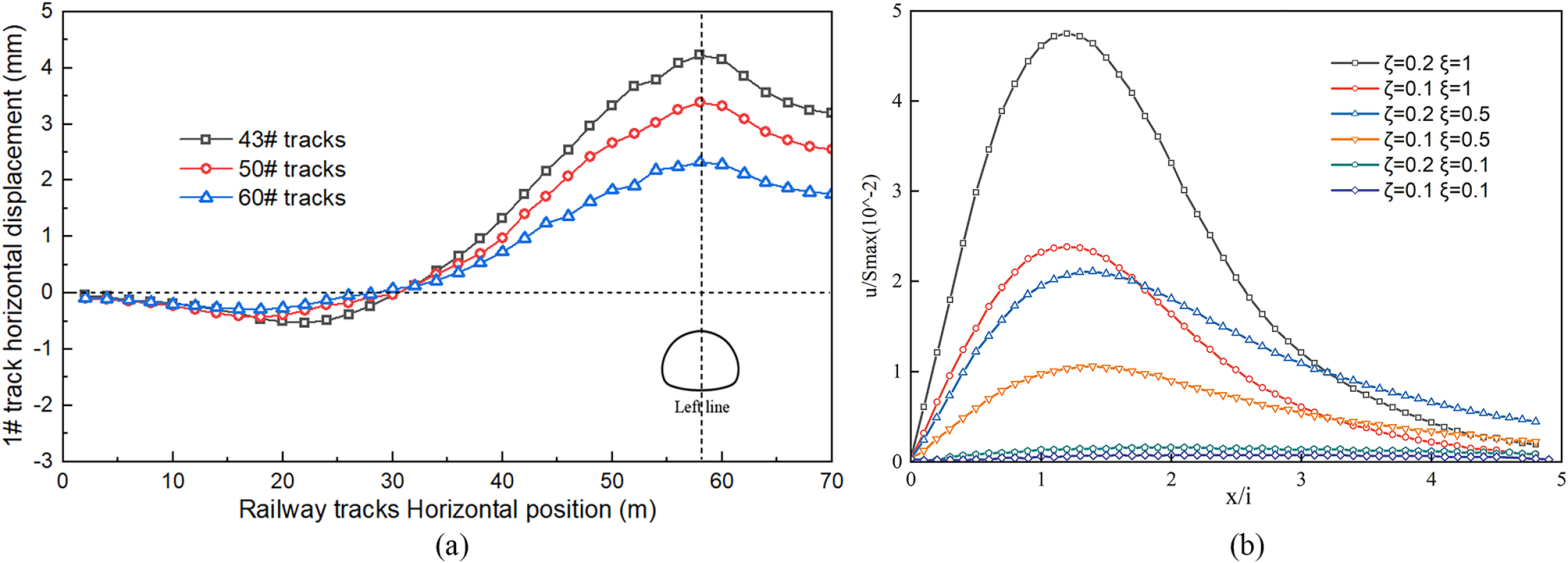
Horizontal displacement changes of different track types: (**a**) horizontal displacement of different track types, (**b**) horizontal displacement changes along the tracks^21^).

Figure 9 illustrates the distribution of the track settlement under various track-type conditions. The change in track settlement differs for different track types, while the surface settlement pattern remains more or less the same. The maximum settlement values for track types 43#, 50#, and 60# are 6.85 mm, 5.67 mm, and 5.13 mm, respectively. The maximum track settlement location is directly above the left line tunnel. The moment of the vertical section and the bending stiffness of the track is directly proportional to the type of track. As the track type and rail bending stiffness increase, the track settlement gradually decreases. The 43# track has the lowest bending stiffness, and its settlement curve is similar to the surface settlement curve. An increase in the rail bending stiffness has some influence on the resistance of the foundation settlement, but this effect is not significant. Figure 10 shows the horizontal deformation of the track caused by tunnel excavation under different track types. The horizontal deformation trends of the different track types are similar. The horizontal displacement of the track caused by tunnel excavation is larger for lower track types. The maximum horizontal displacements for rail types 43#, 50#, and 60# are 4.32 mm, 2.88 mm, and 1.97 mm, respectively. The effect of the ground settlement on track settlement is smaller for more rigid track structures. Zhao Lining and Huang Xiaolin reached similar conclusions in their study of the impact of ground settlement on railway track bed tracks^21^. When the track's flexural stiffness is low, its maximum settlement is close to that of the ground surface. This indicates that the impact of the track flexural stiffness on the track settlement is minimal, and the track settlement is primarily determined by the surface settlement trough. As the relative stiffness of the track to the soil increases, its settlement gradually decreases. This is because the track's resistance to bending deformation increases with increasing soil stiffness.

5 $$ K = \frac{EI}{{\left( {i^{3} r_{0} E_{s} } \right)}} $$

The shape parameters of the settlement ζ and the axial stiffness parameters of the track ξ are introduced. These two parameters are as follows:

6 $$ \zeta = \frac{{\beta i_{z} }}{{z_{m} }} $$

7 $$ {\upxi } = i_{z} \sqrt {\frac{{K_{u} }}{{EA_{b} }}} $$

When ζ is fixed, the absolute value of the maximum horizontal displacement increases with increasing ξ; when x = 5$i_{z}$, the horizontal displacement of the track approaches zero.

### Track differential settlement pattern

**Figure 11:**
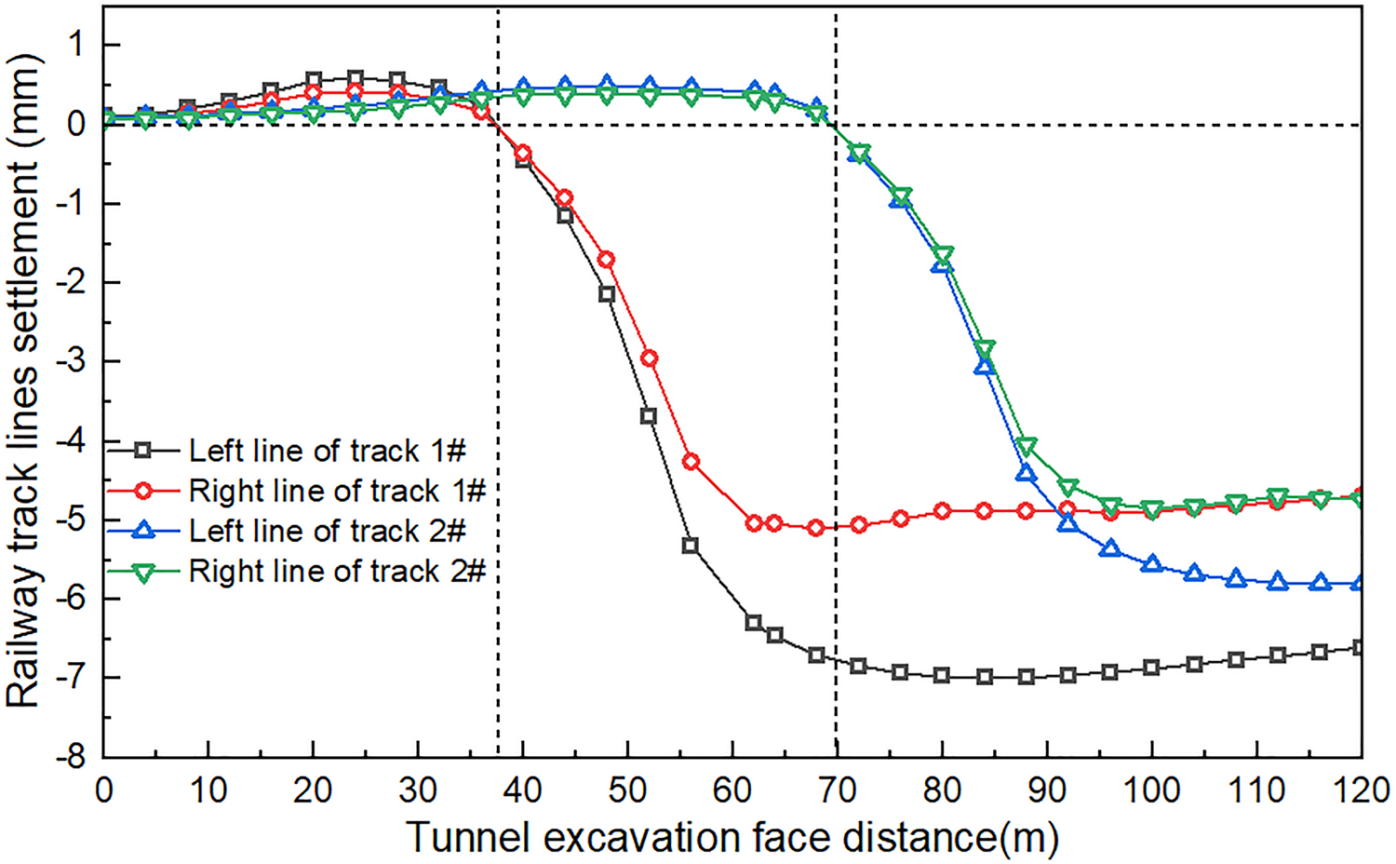
Differential settlement of the track concerning the excavation process.

**Figure 12:**
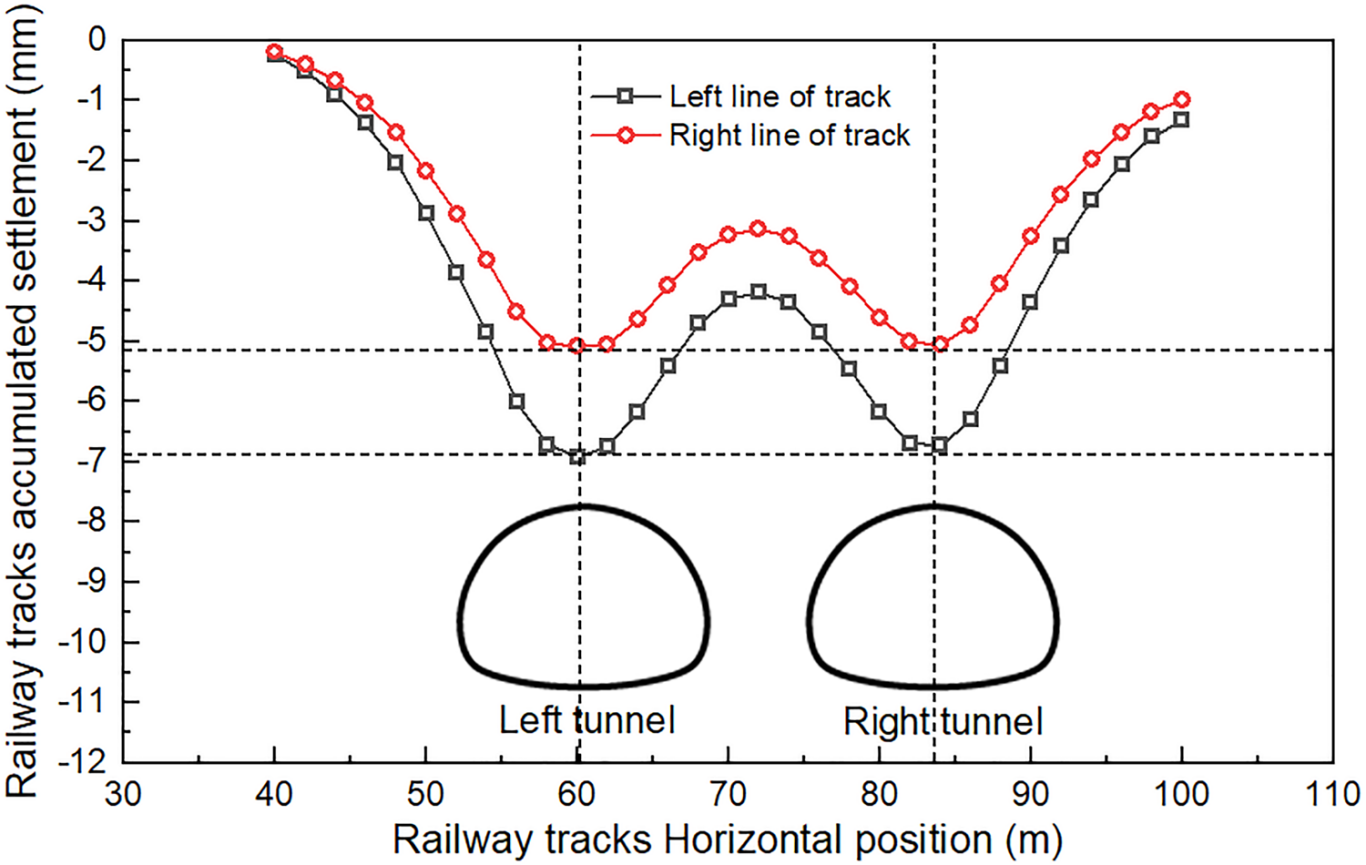
Cumulative differential settlement of the railway double track.

The construction of the tunnel beneath the current railway will cause uneven settlement of the track, resulting in a horizontal height difference between the railway lines. This will cause the top surface of the two rails to be uneven. The unevenness of the track is the primary source of train vibration and increases the force of wheel and rail action, which can significantly impact the safety of train operations. Figure 11 shows the impact of the track-level height difference on the construction process. As the tunnel excavation surface location changes, the settlement of the roadbed and foundation differs for two tracks on the same line. It is important to note that this analysis is objective and does not include any subjective evaluations. When excavating the palm surface between 0 and 30 m, the track experiences varying degrees of augmentation. As the excavation surface reaches 35–75 m, the difference in settlement between the two tracks of Special Line Railway 1 gradually increases with tunnel excavation. By the time the excavation surface reaches 75 m, the track settlement difference gradually converges, and the vertical deviation of the track remains stable. Figure 12 shows the deviation in the horizontal displacement of different tracks. The calculation results indicate that the tunnel construction caused only a small horizontal deviation, with a maximum value of 2.18 mm, which is much smaller than 4. Based on the 00 mm stipulated in the Railway Safe Operation and Management Rules, it is evident that the impact of tunnel construction on the horizontal deviation of the existing railway line is minimal. Proper execution of the track structure during tunnel construction ensures minimal horizontal deviation of the rail track. During tunnel construction, ensuring a proper track structure is crucial for maintaining safe operations. This includes meeting the necessary safety requirements for track-level deviation.

## Control measures

**Figure 13:**
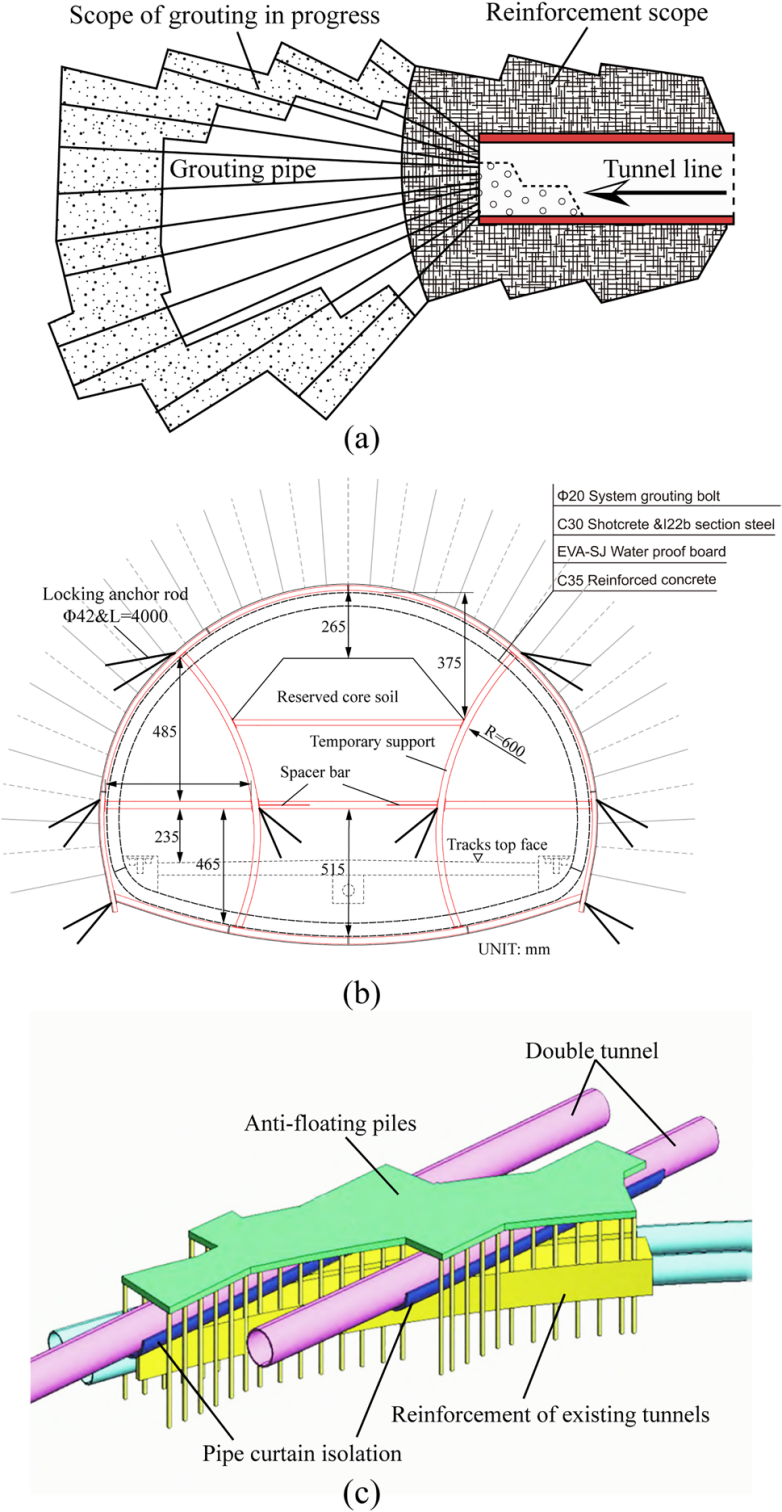
Settlement control measures for tunnel underpass construction: (**a**) grouting reinforcement, (**b**) optimization of excavation method, (**c**) isolation pile wall.

The construction of multiple tunnels beneath a railway is a complex project with many uncertain factors at play. The proximity of the tunnels affects their construction processes, and repeated disturbances in the strata lead to increased subsidence, causing greater deformation of the railway lines and exacerbating track unevenness. To ensure the safe operation of the existing railway, three main measures are currently employed (Fig. 13). First, the foundation soil is reinforced through grouting, improving the integrity of the surrounding rock by improving its physical and mechanical parameters, increasing the grouting pressure, and compensating for the stratum loss. Second, an appropriate excavation method is chosen, the strength of the tunnel support is strengthened, and the construction parameters of the tunnel excavation are controlled to reduce disturbance to the strata during construction. Finally, an isolation pile wall is used to separate the tunnel excavation range and track structure to control the influence degree of tunnel excavation.

**Figure 14:**
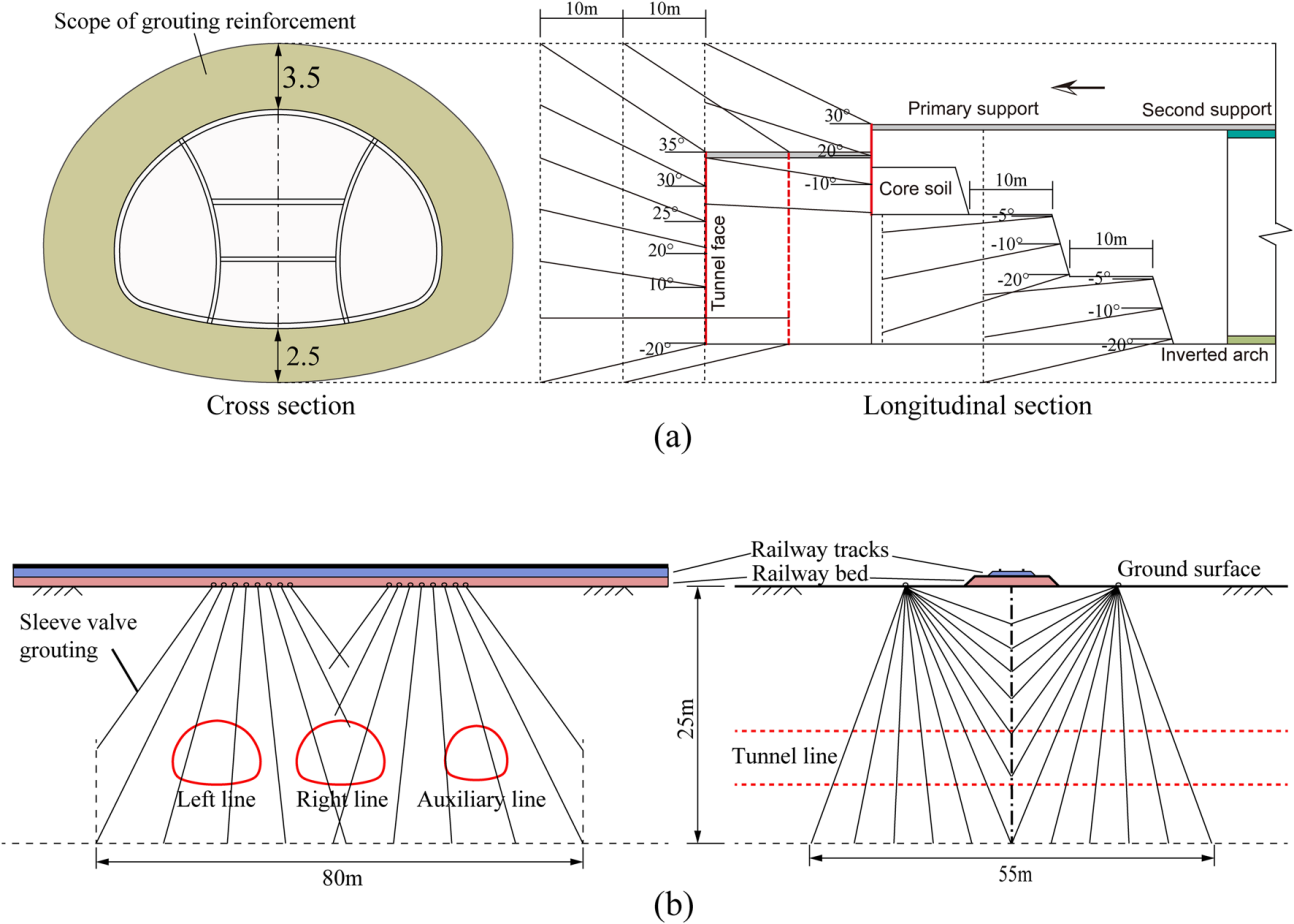
Schematic diagram of tunnel formation reinforcement: (**a**) full-ring grouting of the tunnel, (**b**) grouting of the deep hole in the ground.

This study combines the actual situation of a project, extensive use of perimeter rock grouting, and optimization of construction parameters to evaluate the key parts of large pipe shed construction. By appropriately reinforcing the foundation soil layer under the railway track, the stiffness difference between the tunnel structure and the surrounding soil body is reduced so that the soil stress is evenly distributed. The settlement control method adopts the layer reinforcement method, which combines full-section grouting via holes and soil reinforcement grouting (a layer grouting reinforcement schematic diagram is shown in Fig. 14).

1. The removal length of the temporary elevated arch and the temporary central and adjacent arches should be strictly controlled during construction and appropriately adjusted according to the on-site supervision situation. During construction, the treatment of the arch footing should be reinforced, and 2 Φ42 locking footing anchor tubes with a length of 4 m should be set at the arch footing of each excavation surface and grouted along the whole length. During construction, monitoring and measurement should be intensified, and feedback should be given as soon as possible to guide the construction and correct the support parameters if necessary.
2. The tunnel is reinforced by full-section grouting: the slurry ratio and diffusion radius of full-section grouting are determined according to the test, the grouting pressure is controlled at 0.8–1.0 MPa, the diffusion radius is 0.5 m, and the grouting slurry is a cement–water–glass two-liquid slurry. In addition, according to the conditions under which a layer can be added to adjust the slurry setting time and injectability of additives, grouting water requires a permeability coefficient not greater than 1.0 × 10^−7^ cm/s. The longitudinal length of each section of grouting is 12 m; if there is more than one section of continuous grouting, the length of each section of grouting is 12 m, the length of digging is 10 m, and the lap length is 2 m. For each section of deep hole grouting before the upper step of the core soil area of the palm of the face, a slurry wall is set up. The slurry wall consists of 0.3 m thick C20 shotcrete with double layers of Φ6@150 mm × 150 mm reinforcing mesh and 22@500 mm × 500 mm reinforcing bars. The core floor is protected by 50 mm C20 shotcrete.
3. Ground grouting reinforcement is carried out in the area of the track group before tunneling; for the section reinforced by ground grouting, the unconfined compressive strength of the reinforced soil is at least 0.8 MPa, and the permeability coefficient is at least 1.0 × 10^–^6 cm/s. The borehole is approximately 30 m deep, and the reinforced area is 25 m long and 8.5 m wide. For an empty borehole 14 m below the ground surface, grouting is injected by the sleeve–valve–pipe method, with a borehole diameter of 91 mm, spacing of 500 mm, and diagonal pipe spacing of 400 mm, using a plum blossom arrangement. The radius of the grouting split penetration is 500 mm, the slurry used for cuff pipe grouting is cement slurry, the ratio of slurry water to lime is 1:1, and the grouting pressure is controlled at 0.2–0.5 MPa.

By adopting the double-side drift method with core soil, full-section grouting, and deep hole grouting measures and strictly controlling the construction process, the settlement values all remain within the permissible range, and finally, the goal of controlling the structural settlement of the existing line can be achieved. The research results of this paper have been applied in the studied complex railway underpassing project and have achieved a good control effect. The measures taken provide a successful experience for the construction of similar projects in the future.

## Conclusion

This article studies the effects of longitudinal construction settlement, horizontal cumulative settlement, and differential settlement between tracks on the deformation of a track group when multiple tunnels are constructed underneath a station. The research in this paper leads to the following conclusions.

1. The tunnel construction causes similar deformation patterns in the settlement of the tunnel vault, strata, and tracks. The influence of the settlement trough on the track deformation ranges from 25 to 145 m. The track experiences significant uneven settlement within this range. The section directly above the left tunnel is the location with the greatest impact on multi-tunnel underpass construction.
2. The stiffness of the track can contribute to load dispersion, improving the overall integrity and stability of the track. As the bending stiffness of the steel rail increases, the settlement of the track decreases. The horizontal deformation of the track follows a similar pattern. Uneven tracks are often the result of underpass tunnel construction when the track model is low.
3. When the excavation surface is within the range of 35–75 m, the settlement difference of the railway double track gradually increases. When the excavation surface reaches 75 m, the differential settlement of the track gradually converges and stabilizes. The tunnel construction causes only a small horizontal deviation of the existing railway track, with a maximum of 2.18 mm.
4. To appropriately reinforce the soil layer under the railway track and reduce the stiffness difference between the tunnel structure and the surrounding soil, a combination of full-section grouting from inside the tunnel and reinforcement grouting from the surface is recommended. At the same time, it is recommended to optimize the construction parameters, including step length and primary support closure time.

## Data Availability

The datasets used and/or analyzed during the current study are available from the corresponding author upon reasonable request.

## References

[1] Shahin HMd, Nakai T, Ishii K, Iwata T, Kuroi S (2016). Investigation of influence of tunneling on existing building and tunnel: Model tests and numerical simulations. Acta Geotech..

[2] Li P, Huang H, Qiu W (2020). Investigation of deformation influence on throat area of highway tunnel across the railway station. IOP Conf. Ser. Mater. Sci. Eng..

[3] Fu J, Zhao N, Qu Y, Yang J, Wang S (2022). Effects of twin tunnel undercrossing excavation on the operational high speed railway tunnel with ballastless track. Tunn. Undergr. Space Technol..

[4] Veselý P, Kavička A, Krýže P (2023). Automated construction of mesoscopic railway infrastructure models supporting station throat capacity assessment. IEEE Access.

[5] Zhao X (2022). The settlement law and construction control of railway hubtraversed by a shield tunnel in soft ground. Hazard Control Tunn. Undergr. Eng..

[6] Guo M, Xie J, Song S, Han S (2023). Research on surface settlement induced by construction of new tunnel undercrossing multi-line operating railway. Railw. Investig. Surv..

[7] Klar A, Dromy I, Linker R (2014). Monitoring tunneling induced ground displacements using distributed fiber-optic sensing. Tunn. Undergr. Space Technol..

[8] Liu C, Wang B, Zhou S (2020). Shield tunneling under railway station throats in soft soil areas: A case study. Adv. Civ. Eng..

[9] Ruan L, Sun X, Shen X, Wang S (2018). Research on the impact of new shield tunnel construction on existing railway roadbed. Railw. Stand. Des..

[10] Zhao L, Cai XP, Qu C (2013). Influence of land subsidence on the regularity of the ballastless track with unit slabs. Railw. Stand. Des..

[11] Fattah MY, Shlash KT, Salim NM (2011). Settlement trough due to tunneling in cohesive ground. Indian Geotech. J..

[12] Lv P, Zhou S (2007). Analysis on upper rail settlement law in soft soil ground result from shield tunnelling across main railway line. China Railw. Sci..

[13] Xu G, Li X, Wang H, Zhao Y, Hu P (2009). Analysis of influence of metro shield tunneling crossing underneath high speed railway. Rock Soil Mech..

[14] Liu X (2017). Analysis of influence of subway tunneling under-passing high-speed railway connecting line subgrade. Railw. Stand. Des..

[15] Kavvadas, M. J. Monitoring and modelling ground deformations during tunnelling.

[16] Song Z, Wang K, Wang T, Wang C, Tang S (2019). Analysis of construction method of shallow metro tunnel under the crossing highway. J. Xi’an Univ. Archit. Technol. (Nat. Sci.).

[17] Ocak I (2014). A new approach for estimating the transverse surface settlement curve for twin tunnels in shallow and soft soils. Environ. Earth Sci..

[18] Bian X, Jiang H, Chang C, Hu J, Chen Y (2015). Track and ground vibrations generated by high-speed train running on ballastless railway with excitation of vertical track irregularities. Soil Dyn. Earthq. Eng..

[19] Wang Z (2019). Analysis of ground surface settlement induced by the construction of a large-diameter shallow-buried twin-tunnel in soft ground. Tunn. Undergr. Space Technol..

[20] China Railway Publishing House. The standard of China railway line repair rules (2006).

[21] Huang X, Zhou Z, Liu Q (2019). Study on internal forces and deformation of the high-speed rail by the excavation of the shallow tunnel. J. Hunan Univ. Sci. Technol. (Nat. Sci. Ed.).

